# Impact of Peer-Led Simulation-Based Medical Education on Knowledge and Confidence in Managing Acutely Ill Patients: A Pilot Study Among Pre-clinical Medical Students

**DOI:** 10.7759/cureus.94464

**Published:** 2025-10-13

**Authors:** Ashwin Anand, Chibuchi Amadi-Livingstone, Rishen Cattaree, Joanne Harris

**Affiliations:** 1 Department of Surgery, Monash Health, Melbourne, AUS; 2 Department of Health Sciences, University of York, York, GBR; 3 Department of Anaesthesiology, Milton Keynes University Hospital NHS Foundation Trust, Milton Keynes, GBR; 4 Department of Medical Education, University of East London, London, GBR

**Keywords:** acute illness management, confidence, knowledge, near-peer teaching, simulation

## Abstract

Background

Preparedness to manage acutely ill patients is a critical competency for new medical graduates, yet there are persisting gaps in acute-care management teaching throughout undergraduate training. To address this, we developed the Systematic Approach for the Acutely ill Patient (SAAP) course, a peer-led, simulation-based medical education (SBME) programme designed to give pre-clinical medical students a solid foundation in acute illness management. The course aimed to improve their knowledge and confidence in managing acutely ill patients, thereby better preparing them for the transition into clinical training.

Methods

This pilot quasi-experimental study employed a pre- and post-test design involving 70 pre-clinical medical students at a UK university. The SAAP course comprised four workshops based on the RATE framework (Recognise, Assess, Treatment, Escalation), followed by simulation scenarios facilitated by near-peer instructors. Students completed knowledge tests and confidence questionnaires before and after the simulation and provided qualitative feedback at the end of the course. Data were analysed using paired t-tests for knowledge scores and Mann-Whitney U tests for questionnaire responses.

Results

There was a significant increase in knowledge score across the study cohort (mean pre-test score 20.36 to post-test 22.17, p < 0.001). First-year students (19.03 to 21.08, p < 0.001) demonstrated the larger improvement compared to second-year students (22.03 to 23.55, p < 0.001). Confidence scores rose significantly in multiple domains, including ordering appropriate investigations (3.24 to 3.83, p < 0.001), managing patients with breathing difficulties (3.46 to 3.91, p < 0.001), managing hypotension (3.50 to 3.89, p < 0.00), and using the Identify, Situation, Background, Assessment, Recommendation (ISBAR) framework for escalation (3.77 to 4.06, p = 0.004). Overall confidence in acute-care management improved from 3.46 to 3.86 (p < 0.001). Qualitative feedback highlighted the value of near-peer facilitation, the clinical relevance of scenarios, and the psychologically safe learning environment that near-peer educators provided.

Conclusions

A peer-led acute-care simulation course significantly improved both knowledge and confidence among pre-clinical medical students. The findings suggest that vertically integrated, near-peer SBME can bridge the gap between theory and clinical practice, preparing students for acute-care responsibilities earlier in training. While immediate gains were clear, further longitudinal research is needed to assess retention, scalability, and comparative effectiveness versus faculty-led approaches.

## Introduction

Training on diagnosis and management of acute medical emergencies is essential for medical students [1], and the General Medical Council (GMC) mandates that newly qualified doctors must be able to recognise when a patient is deteriorating and take appropriate action [2]. A recent report found that newly graduated doctors were not prepared for complex clinical decision-making in acute settings and concluded that training in this area requires more attention [3]. Other studies have also identified this lack of preparedness from undergraduate medical students in managing acute patients, with one systematic review concluding evident deficiencies in recognition and management of acutely unwell patients [4,5]. Training is a key issue in the early detection and management of acutely ill patients [6]. Despite the theoretical aspects being taught during undergraduate training, there are concerns from both newly qualified doctors and their clinical supervisors surrounding preparedness within the real-life clinical context [7].

During our penultimate year at medical school, we observed a hesitation amongst ourselves and our peers when encountering acutely unwell patients. While we felt confident in assessing stable patients, we often lacked the knowledge, structure, and confidence to fully understand and anticipate the clinical management steps taken by senior doctors when patients were deteriorating. Discussions with fellow students confirmed that this was a common concern, highlighting a gap between our theoretical learning and the realities of acute care environments. For this reason, we designed the Systematic Approach for the Acutely ill Patient (SAAP) course, which utilises a near-peer simulation-based approach to provide pre-clinical medical students with a solid foundation for the care of acutely ill patients. A near-peer can be defined as a learner who is one year or more senior to another learner undertaking the same training [8].

Practice of these skills within a clinical setting for undergraduate medical students is restricted by limitations on their permitted activities [9]. Students may only practise unfamiliar skills under the direct supervision of doctors or other healthcare professionals who are competent in performing them. However, staffing pressures in hospitals can make this difficult, limiting students’ opportunities to develop these skills. To bypass this, simulation has become a popular approach for teaching the management of the acutely unwell patient. This is because it promotes teamwork and offers the opportunity for learners to experience a scenario which is similar to a real-life event but without a threat to patient safety [10-12]. Many studies have found near-peer teaching to be popular and effective amongst students [13-15]. Near-peer teaching works on principles of cognitive and social congruence [16,17]. Cognitive congruence suggests that since teachers and learners share a similar knowledge base, this enables teachers to better empathise with the learners' perspective compared to faculty-experts. They can draw on their own recent learning experiences to explain concepts, anticipate common challenges, and prevent cognitive overload by avoiding unnecessary detail. Additionally, near-peer teachers benefit from social congruence, as they hold a comparable position within the medical journey. This fosters a more comfortable and supportive learning environment, encouraging an open dialogue and the free exchange of ideas [16]. The SAAP course introduced students to the basic principles of detecting clinically deteriorating patients, taking prompt action to stabilise those patients, and familiarising students with the management of common acute conditions. By introducing this early in their training, we aimed to improve students’ understanding of acute-care patient management, build confidence, and better prepare them for their clinical years. This study aims to evaluate the efficacy of simulation-based medical education (SBME) on the knowledge and self-reported confidence of pre-clinical medical students in managing acutely ill patients.

This article was previously presented as a meeting abstract at the 2025 ASME Annual Scholarship Meeting on July 2, 2025.

## Materials and methods

Study design and setting

**Figure 1:**
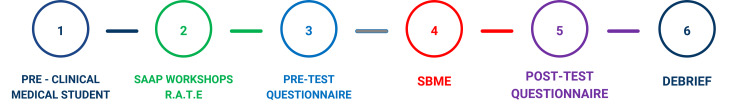
Diagram showing how the SAAP session was organised during the study SAAP: Systematic Approach for the Acutely ill Patient; SBME: simulation-based medical education; RATE: Recognise, Assess, Treatment, Escalation Image credit: The authors

This pilot study utilised a quasi-experimental pre-test and post-test design. A questionnaire and knowledge test were used to assess the effectiveness of near-peer SBME on the confidence and knowledge of pre-clinical medical students in managing acute illnesses. To uphold educational equity and ethical responsibility, this study does not contain a control group but rather analyses changes within the same student cohort before and after the intervention. On the day, students first participated in the SAAP teaching workshop, which was facilitated by trained peer instructors who were at least two academic years ahead of them. This is because, at our institution, students complete an acute patient management course during their third year. Successfully passing this course demonstrates that they have developed foundational competence in managing acutely unwell patients. As a result, they are considered capable of teaching their junior colleagues, provided they receive adequate guidance. Following the workshops, there was a 10-minute break before students completed the pre-test, which assessed their baseline knowledge, and the questionnaire, which evaluated their baseline self-reported confidence. Thereafter, students immediately participated in two back-to-back simulation scenarios facilitated by peer instructors. After completing these scenarios, students completed the knowledge post-test and questionnaire to assess changes in their knowledge and self-reported confidence following the simulations (Figure 1). The pre-test was administered after the workshops and prior to the simulation scenarios, as the primary aim was to assess the impact of the simulation-based learning experience on students’ knowledge and confidence. The workshops were designed to ensure students had sufficient foundational knowledge to engage meaningfully in the simulations. To avoid measurement bias that could arise from differences in question difficulty and ensure direct comparability of data, the same questions were used in the pre-test and post-test. Students were not informed that the pre-test and post-test contained identical questions until they received the post-test paper, and test feedback was only given after the post-test. This was done to prevent collusion during the simulation session and prevent students from attempting to memorise specific questions. Simulation scenarios also began immediately after the pre-test, preventing students from having an opportunity to discuss test questions. Facilitators had no knowledge of the test questions or the fact that the pre-test and post-test were identical. This was designed to eliminate potential bias, ensuring that facilitators provided neutral guidance during the simulation without inadvertently influencing students’ performance on the post-test.

Participants and setting

The study was carried out over six SAAP sessions delivered to first- and second-year medical students at the University of Buckingham Medical School. The sessions were completed by first-year medical students towards the end of their first year and second-year students midway through their second year. The purpose was to ensure that all students had undertaken university teaching on clinical examination, anatomy and pathophysiology of the major body systems, thereby meeting the minimum requirements to participate effectively in the course. The setting of this study was the clinical skills rooms of the University of Buckingham Medical School. The University operates a 4.5-year programme based on the outcomes and standards for undergraduate medical education stated in the GMC publication “Outcomes for Graduates (2018)” [2]. The medical school operates a longitudinal curriculum, combining systems-based learning with early clinical exposure in the first two years, followed by 2.5 years of clinically focused teaching and placements.

SAAP course development and design

**Figure 2:**
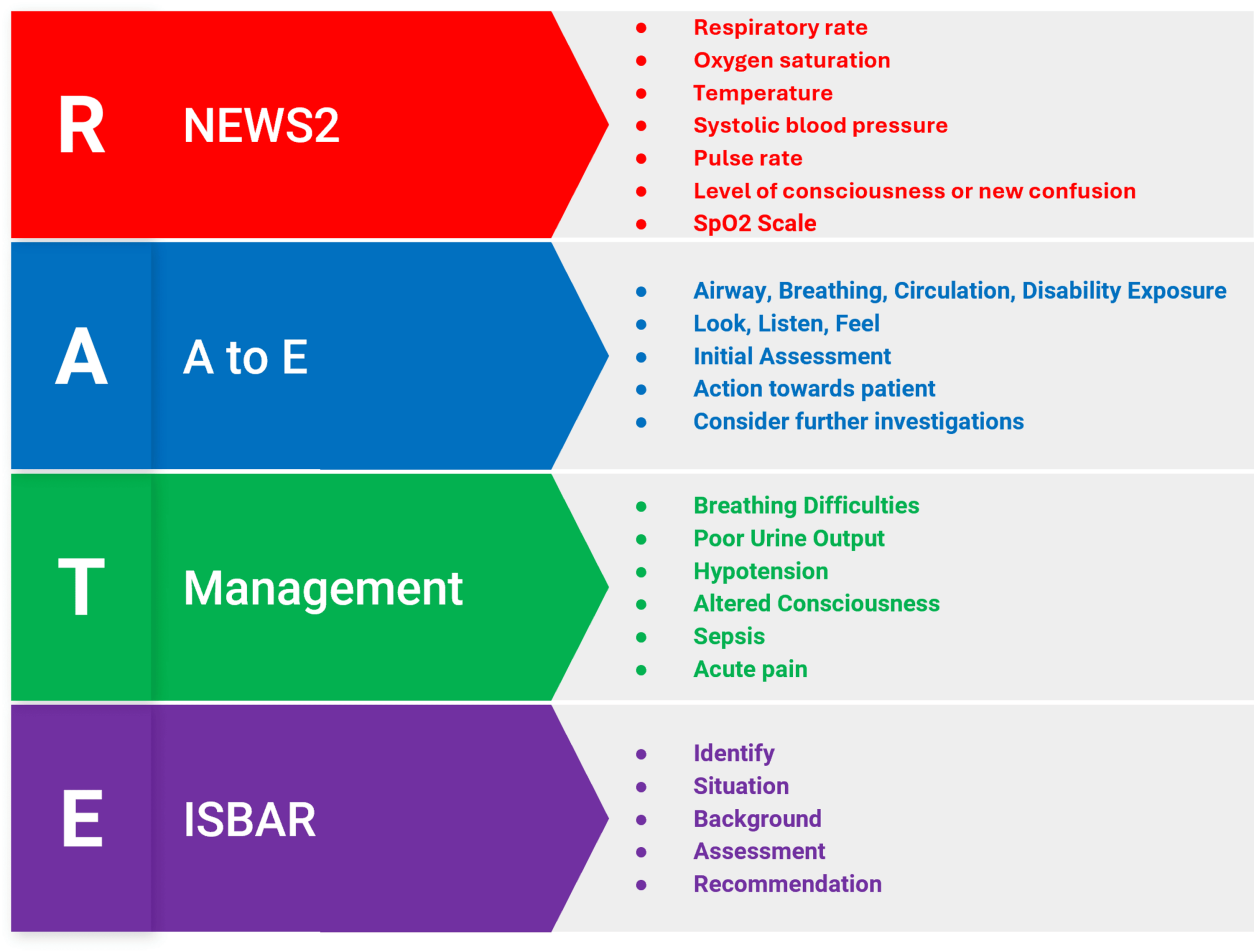
The SAAP core principles (Recognise, Assess, Treatment, Escalation) SAAP: Systematic Approach for the Acutely ill Patient; NEWS2: National Early Warning Score 2; A to E: Airway, Breathing, Circulation, Disability, Exposure; ISBAR: Identify, Situation, Background, Assessment, Recommendation Image credit: The authors

The aim of the SAAP course is to develop the clinical confidence of pre-clinical medical students in acute-care management. The course was an optional programme for pre-clinical medical students, created and delivered by senior medical students, and participation took place outside of mandatory curricular time. The course was structured around four key themes: Recognise, Assess, Treatment and Escalation (RATE). In Recognise, students are taught to identify acutely ill patients using the National Early Warning Score 2 (NEWS2) scoring system [18], which evaluates physiological parameters such as respiratory rate, systolic blood pressure and oxygen saturation. In Assess, students are taught to systematically review patients using a structured Airway, Breathing, Circulation, Disability, Exposure (A-E) approach and carry out immediate interventions where necessary. The A-E approach is a structured, systematic method used in healthcare to assess and manage acutely unwell patients. In Treatment, students are introduced to general guidance for management of common acute conditions such as breathing difficulties, hypotension, sepsis, altered consciousness level, acute kidney injury and acute pain. Students then learn how to Escalate care using the Identify, Situation, Background, Assessment, Recommendation (ISBAR) framework, teaching them how to handover care in a safe and structured way, an important aspect of acute-care management. During the course, the themes are broken into four workshop stations, which are taught by trained near-peer tutors (Figure 2). Each tutor is responsible for delivering a dedicated workshop station according to our developed course material, with students rotating through all four stations. Following these workshops, students are divided into small groups and take part in two to three simulated acute-care scenarios delivered by peer facilitators. Each simulation focuses on managing one acutely ill patient with a specific medical condition. Students are expected to utilise the principles of RATE in managing these patients, and all feedback is delivered according to each of the four domains. After the simulation, students take part in a 10-minute debrief session, allowing them to reflect on their performances, address challenges faced, discuss learning points and provide emotional support to students who may require it. Peer tutors completed a training session led by the course creators. During the session, tutors were instructed on how to deliver the RATE teaching workshop with a focus on promoting learner engagement and psychological safety. They were trained to manage session timing, facilitate each component of the workshop, and guide simulations effectively. Particular emphasis was placed on strategies for delivering cues, maintaining fidelity, and applying structured feedback. To support their teaching, peer tutors were also provided with the course manual, simulation feedback guide, and slide decks.

Knowledge test and questionnaire

The knowledge test was designed to evaluate students’ understanding and application of basic principles taught during the course. It evaluated students’ ability to apply the RATE framework in acute-care scenarios. It consisted of 30 single best answer questions, with nine evaluating assessment, nine on treatments, six on recognition and six on escalation.

The questionnaire was designed to measure the students’ confidence in managing acutely unwell patients and in utilising the skills and principles taught during the course. The questionnaire consisted of 10 items measured on a Likert scale. Each item is scored on a scale of 1 to 5, where 5 represents “strongly agree”, 4 represents “agree”, 3 represents “neutral”, 2 represents “disagree”, and 1 represents “strongly disagree”. The knowledge questions and questionnaire items were content validated by a senior university lecturer and a specialist anaesthetist. In terms of reliability, the Cronbach alpha scores for pre- and post-simulation confidence questionnaires were 0.850 and 0.891, respectively, indicating good levels of internal consistency. The tests and questionnaire were distributed to students on A4 sheets of paper, with a maximum test completion time of 25 minutes.

Statistical analysis

IBM SPSS Statistics for Windows, Version 29 (Released 2021; IBM Corp., Armonk, New York, United States) was used for all statistical analyses. A paired t-test was used to compare the differences in students’ performance between the pre-simulation and post-simulation knowledge tests. Since Likert scale responses were deemed non-normally distributed based on the Shapiro-Wilk test, the non-parametric Wilcoxon Signed-Rank test was used to compare responses between the pre-simulation and post-simulation questionnaires. The Cronbach alpha test for reliability was used to measure the internal consistency of the questionnaire items. A p-value of < 0.05 was considered statistically significant.

## Results

Knowledge test

All 70 students who participated in the study submitted completed questionnaires and test papers. Of these, 39 were first-year students and 31 were second-year students. The mean pre-test and post-test scores, along with their standard deviations (SDs), are presented in Table 1. The overall pre-test score is 20.36 (SD = 3.27). When stratified by year group, second year students achieved a higher mean pre-test score of 22.03 (SD = 2.79) compared to the 19.03 (SD = 3.02) achieved by first year students. The post-test saw a statistically significant improvement in the score for all students (mean = 22.17, SD = 3.19, p < 0.001). There was also a statistically significant increase in post-test scores for first and second years as their mean scores improved to 21.08 (SD = 3.21, p < 0.001 and 23.55 (SD = 2.62, p < 0.001), respectively. The improvement in overall knowledge was associated with a large effect size (d = -0.89). Both year groups also showed large effects: first years (d = -0.90) and second years (d = -0.93), indicating that the observed gains were not only statistically significant but also educationally meaningful.

**Table 1:** Results of the pre- and post-simulation knowledge tests for first year (Y1), second year (Y2), and combined (Y1 + Y2) The paired t-test was used to compare responses between the pre-simulation and post-simulation questionnaires. * A p-value < 0.05 was considered statistically significant. Effect size (Cohen’s d) represents the standardised mean difference, with ±0.2 = small, ±0.5 = medium, and ±0.8 = large effect.

Group	Pre-test, mean (SD)	Post-test, mean (SD)	Difference	P-value	T-statistic	Effect size (Cohen’s d)
Y1 + Y2 (n = 70)	20.36 (3.27)	22.17 (3.19)	1.81	<0.001*	-7.47	-0.89
Y2 only (n = 31)	22.03 (2.79)	23.55 (2.62)	1.52	<0.001*	-5.18	-0.93
Y1 only (n = 39)	19.03 (3.02)	21.08 (3.21)	2.05	<0.001*	-5.59	-0.9

Confidence questionnaire

**Figure 3:**
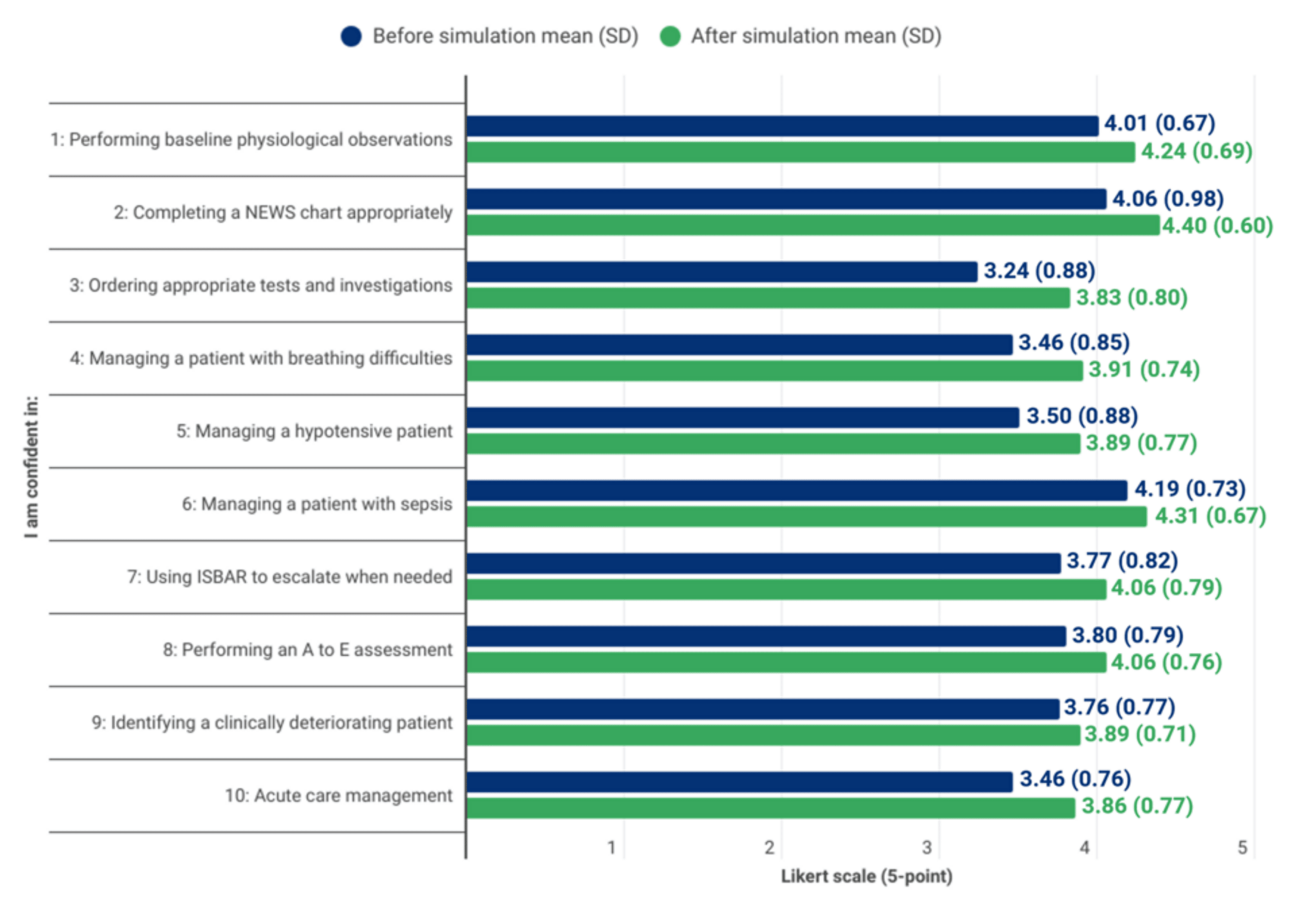
Graphical representation of the results for pre- and post-simulation confidence questionnaire for all students NEWS2: National Early Warning Score 2; A to E: Airway, Breathing, Circulation, Disability, Exposure; ISBAR: Identify, Situation, Background, Assessment, Recommendation

Table 2 and Figure 3 show the mean confidence scores and SDs for each questionnaire item, before and after the simulations. There was an increase in mean confidence scores for all items following the simulation sessions. The greatest improvement in the mean self-reported confidence was observed in ordering appropriate tests and investigations, which also showed a statistically significant rise from 3.24 (SD = 0.88) to 3.83 (SD = 0.80) (p < 0.001). Notably, this was also the area in which students reported the lowest confidence before the simulation. In contrast, prior to the simulations, students reported the most confidence in the management of a patient with sepsis. However, this area showed the smallest improvement in scores from 4.19 (SD = 0.73) to 4.31 (SD = 0.67) and did not achieve a statistically significant change post-simulation (p = 0.097). Whereas other areas involving the management of acute clinical problems, such as managing a patient with breathing difficulties (from 3.46 to 3.91, p < 0.001) and managing a hypotensive patient (from 3.50 to 3.89, p < 0.001), demonstrated statistically significant improvements. Further areas that demonstrated a statistically significant increase in self-reported confidence following the simulations included appropriate completion of a NEWS chart (from 4.06 to 4.40, p = 0.006), performing baseline physiological observations (from 4.01 to 4.24, p = 0.008), and using ISBAR to escalate when needed (from 3.77 to 4.06, p = 0.004). Despite an increase in mean self-reported confidence following the simulations, confidence in performing an A to E assessment (from 3.80 to 4.06, p = 0.180) and in identifying a clinically deteriorating patient (from 3.76 to 3.89, p = 0.180) did not show statistically significant changes. Overall, confidence in acute-care management showed statistically significant improvement, with mean scores increasing from 3.46 (SD = 0.76) before the simulations to 3.86 (SD = 0.77) following the simulations (p < 0.001). Confidence improvements ranged from small to large effects across domains. A strong effect size was seen in confidence in ordering appropriate tests and investigations (r = -0.42). Indication of a strong association between participating in simulations and an increase in self-reported confidence. Medium effect sizes were observed in self-reported confidence in managing breathing difficulties (r = -0.36), hypotension (r = -0.33) and overall acute care management (r = -0.33). We observed small effect sizes in the other domains for self-reported confidence, suggesting a weak association between simulation and self-reported confidence gains in those areas.

**Table 2:** Results for pre- and post-simulation confidence questionnaire for all students The Wilcoxon signed-rank test was used to compare responses between the pre-simulation and post-simulation questionnaires. * A p-value < 0.05 was considered statistically significant. Effect size (r) represents the strength of association calculated as Z/√N, where ±0.1 = small, ±0.3 = medium, and ±0.5 = large effect. NEWS2: National Early Warning Score 2; A to E: Airway, Breathing, Circulation, Disability, Exposure; ISBAR: Identify, Situation, Background, Assessment, Recommendation

I am confident in:	Before simulation, mean (SD)	After simulation, mean (SD)	P-value	Z-value	Effect size (r)
					r= Z/√N
1. Performing baseline physiological observations	4.01 (0.67)	4.24 (0.69)	0.008*	-2.67	-0.23
2. Completing a NEWS chart appropriately	4.06 (0.98)	4.40 (0.60)	0.006*	-2.77	-0.23
3. Ordering appropriate tests and investigations	3.24 (0.88)	3.83 (0.80)	<0.001*	-4.92	-0.42
4. Managing a patient with breathing difficulties	3.46 (0.85)	3.91 (0.74)	<0.001*	-4.3	-0.36
5. Managing a hypotensive patient	3.50 (0.88)	3.89 (0.77)	<0.001*	-3.91	-0.33
6. Managing a patient with sepsis	4.19 (0.73)	4.31 (0.67)	0.097	-1.66	-0.14
7. Using ISBAR to escalate when needed	3.77 (0.82)	4.06 (0.79)	0.004*	-2.86	-0.24
8. Performing an A to E assessment	3.80 (0.79)	4.06 (0.76)	0.18	-2.37	-0.2
9. Identifying a clinically deteriorating patient	3.76 (0.77)	3.89 (0.71)	0.18	-1.34	-0.11
10. Acute-care management	3.46 (0.76)	3.86 (0.77)	<0.001	-4	-0.33

Student feedback

Throughout the study, feedback on the course was routinely collected from the students at the end of each session. Free-text feedback comments were received from 51 participants. The comments were analysed using thematic techniques and the following themes arose: Comments on the value of near-peer teaching, enthusiasm of facilitators, clinical relevance and organisation of the course (Table 3).

**Table 3:** Themes of free-text feedback with examples OSCE: Objective Structured Clinical Examination

Theme	Example statement
1. Value of near-peer teaching	“The 4th years were very engaging, passionate and patient when they taught the session. Loved it”
	“Interacting with phase 2 students and learning about the clinical relevance of the course material was really helpful”
2. Engagement and enthusiasm of facilitators	“Very interactive and engaging. Enthusiastic approach and an extremely comfortable environment in which to learn”
	“I feel as though the entire course was delivered with utmost resilience, motivation, hard work and passion from the team. Everyone was welcoming, engaging and informative”
	“It was a great learning atmosphere and also a space to have fun as well”
3. Practical and clinical relevance	“The content was highly relevant and meaningful since it helped establish theoretical and clinical connections. Having to deal with case scenarios like this helps us prepare for our OSCEs and clinical placements”
	“The application of content learnt so far into a clinical situation that is time-sensitive was shocking to the system in the best way”
4. Structure and organisation of the course	“The structure of the course was logical, having the simulation after the teaching to put it all into practice”
	“The structure, delivery and organisation of the course was excellent and well mirrored the content we learnt throughout phase 1”

## Discussion

Participation in a concise, near-peer acute-care simulation course yielded immediate gains in both knowledge and self-confidence among our set of pre-clinical medical students. Knowledge gains were consistent across both years, with greater absolute improvement among first-year students, and confidence increased in key clinical domains.

Qualitative feedback indicated that pre-clinical students valued applying clinical context to bridge theory and practice. They saw simulations as a useful way to prepare for placements by developing their skills in clinical assessment and management. Students valued the opportunity to practise these skills in a comfortable, supportive environment facilitated by enthusiastic peer tutors.

These findings indicate that a brief, focused simulation curriculum delivered by near-peer instructors is associated with measurable improvements in pre-clinical students’ preparedness for acute care.

Knowledge

In the primary knowledge outcome, students demonstrated significant improvement immediately after the simulation. On the 30-item assessment, cohort mean scores increased from 20.36 (SD 3.27) to 22.17 (SD 3.19), a gain of 1.81 points (p < 0.001). Both year groups improved: year 1 rose from 19.03 (SD 3.02) to 21.08 (SD 3.21) and year 2 from 22.03 (SD 2.79) to 23.55 (SD 2.62) (both p < 0.001). The greater absolute improvement among year 1 students, who began at a lower baseline, indicates that learners early in training can consolidate core acute-care concepts when these are rehearsed in realistic scenarios. These findings indicate that students at an early stage can rapidly assimilate acute-care concepts when taught through targeted simulations [19]. The pattern reflects a consistent, cohort-wide shift in knowledge immediately after the session.

These findings should be interpreted in the context of several methodological considerations. The numerical gain is modest on a 30-point scale and is best viewed as short-term consolidation rather than mastery. Reuse of identical pre- and post-items may have introduced a testing effect, and administering the pre-test after workshops cannot entirely exclude residual teaching influence. Outcomes were measured immediately after the session, so durability is unknown. The pre-test was scheduled after the workshops to isolate the incremental effect of the simulation; however, this sequencing may permit residual teaching influence. Single-site delivery and reliance on trained near-peer facilitators may limit generalisability.

Confidence

Confidence improved across multiple domains. Overall acute-care confidence rose from 3.46 (SD 0.76) to 3.86 (SD 0.77; p < 0.001). The largest gain was in ordering appropriate tests and investigations (3.24 to 3.83; p < 0.001). Confidence also increased for managing breathing difficulties (3.46 to 3.91; p < 0.001) and managing hypotension (3.50 to 3.89; p < 0.001), with additional improvements in completing a NEWS chart (4.06 to 4.40; p = 0.006), performing baseline observations (4.01 to 4.24; p = 0.008), and using ISBAR to escalate (3.77 to 4.06; p = 0.004). Smaller, non-significant changes were observed for sepsis management (4.19 to 4.31; p = 0.097), A to E assessment (3.80 to 4.06; p = 0.180), and identifying a clinically deteriorating patient (3.76 to 3.89; p = 0.180), which likely reflects high starting confidence and the need for repeated practice in these complex domains [20]. These shifts indicate a broader rise in perceived readiness and align with qualitative feedback highlighting authentic clinical context, preparation for placements and a supportive learning environment led by engaged peer tutors.

These findings reflect self-reported confidence rather than observed performance and were captured immediately post-session, so durability is unknown and short-term perceptions may be influenced by novelty or social desirability. Confidence appears to rise most when learners can practise discrete decision points and receive immediate reinforcement, whereas multi-step cognitive bundles show less movement after a single session. Educational implications include sequencing scenarios to revisit complex domains at progressively higher levels of difficulty, integrating brief retrieval and rehearsal activities between sessions, and aligning debrief prompts with specific escalation and assessment behaviours. Future evaluations should pair confidence outcomes with objective performance measures and incorporate delayed follow-up to determine whether perceived gains are sustained and translate into observable improvement in clinical tasks.

Simulation-based medical education (SBME)

SBME represents a particularly effective platform for introducing novice learners to the cognitive and procedural demands of acute-care practice [21]. Within the SAAP programme, simulation serves as the pedagogical core, allowing students to rehearse the RATE framework. Such immersive yet low-risk environments are widely acknowledged as ideal for acute-care instruction because they mirror the time pressure and complexity of real emergencies while preserving learner and patient safety [9-11].

SBME is repeatedly linked to improved procedural skill, durable knowledge and enhanced team performance, and it now underpins most undergraduate deteriorating-patient programmes [22]. A 2023 survey of 139 US medical schools found nearly every curriculum now embeds simulation [23]. Learners in the present study likewise judged the scenarios to be highly authentic, a perception that likely reinforced both knowledge acquisition and self-efficacy. Additionally, structured debriefing, which is considered a best practice in SBME, allows for reflection on errors and successes, further consolidating learning [24].

Even at the preclinical level, early exposure to simulation accelerates readiness for clinical rotations, allowing students to participate actively rather than remain passive observers [25]. The GMC stipulates that new graduates must “recognise and manage acutely ill patients” [2]. Investment in simulation infrastructure and faculty or near-peer tutor development, therefore, represents a tangible means of meeting the mandated graduate outcomes.

Near-peer facilitation

A distinctive element of SAAP is the use of near-peer facilitators. This model proved both feasible and highly effective within the simulation setting; peer instructors conveyed complex acute-care concepts in language that resonated with first- and second-year learners, yielding substantial knowledge gains. The effectiveness of this approach can be attributed to cognitive congruence. As tutors were only slightly further advanced in their training, they were able to anticipate the misconceptions of novice learners and tailor their explanations appropriately. Additionally, social congruence played a key role in creating a psychologically safe environment. This encouraged questioning and allowed for learning through error [26].

Near-peer simulation not only benefits junior learners but also strengthens tutors’ own skills, emphasising its reproducibility [27]. Its impact stems chiefly from fostering a collegial atmosphere and supplying supportive resources that make learning a positive experience [28]. Beyond immediate learning gains, near-peer programmes cultivate teaching acumen and leadership in senior students [29]. Participants in the present course echoed these advantages, describing a “safe and protected” space that encouraged active management of simulated patients and heightened readiness for forthcoming clinical duties. Other research highlights that peer-led teaching can be as effective as faculty-led simulation for selected skills, with added benefits of cost efficiency and tutor development [30]. This dual benefit makes them a useful complement to faculty curricula.

In near-peer programmes, several limitations warrant consideration. Tutor professionalism and engagement vary; some arrive late, prepare inadequately, or deviate from objectives. Preparation and delivery require substantial time for material development, rehearsal, and coordination, which competes with academic study and intensifies during examination periods, increasing stress and the risk of burnout. Complex questions may exceed tutor expertise, resulting in delays or incomplete answers [31]. A persistent risk is inaccurate teaching and drift from curricular standards. To mitigate these issues, faculty should provide regular booster sessions, supply standardised materials and checklists, and establish clear escalation routes for unresolved queries, thereby preserving accuracy and alignment with objectives. Embedding structured near-peer simulation provides a scalable, cost-effective mechanism to achieve acute-care competence while simultaneously cultivating the next generation of clinical educators [32].

Curriculum integration and vertical alignment

Early incorporation of simulation affords staged exposure to increasingly complex acute-care scenarios well before students face them in high-stakes clinical environments. Furthermore, synchronising these exercises with concurrent physiology and pharmacology maximises contextual relevance and knowledge retention in line with spiral-curriculum theory [33]. In doing so, the programme puts into practice the principle of vertical integration, and this educational philosophy promotes steady learner growth and deeper professional engagement [34].

Embedding acute-illness management early in the curriculum exemplifies this approach, where foundational science is revisited in a progressively richer clinical context. For instance, a student who encounters a simulated patient with refractory hyperaemia can link the observed desaturation to shunt physiology and the underlying mechanism of respiration, thereby transforming abstract concepts into actionable clinical reasoning. Empirical work indicates that physiology taught through vertically integrated, case-based approaches generates greater engagement and deeper, more durable understanding than conventional, discipline-siloed instruction curricula [35]. Thus, staged exposure to acute-care scenarios not only rehearses practical algorithms but also consolidates core scientific principles, fostering meaningful learning that persists into later training [36]. Vertically integrated simulation constructs a resilient cognitive-practical scaffold that learners can confidently deploy when confronted with real-world patient deterioration.

Knowledge retention and decay

Despite the robust immediate gains observed, the longevity of these effects remains undetermined. Outcomes were recorded only at baseline and immediately post-intervention; thus, the extent to which knowledge persists over subsequent weeks or months is unknown. According to Ebbinghaus’ classical model of the forgetting curve, unrehearsed learning tends to decline rapidly over time without reinforcement [37]. This process is likely intensified by the substantial cognitive load associated with medical training.

Consequently, the benefit of a single session of simulation may diminish without systematic reinforcement, and essential acute-care skills such as structured A to E assessment and effective escalation using the ISBAR framework may erode before students enter their clinical years. The lack of a delayed post-test in the study design prevents assessing if learning may have decayed over time.

Encouragingly, a recent study found no significant score drop up to three months post simulation, suggesting that well-designed scenarios and debriefs can anchor learning [38]. Nonetheless, longitudinal booster sessions or spaced repetition approaches are advisable to mitigate decay [39,40]. Embedding such refreshers at key transition points could convert SAAP’s short-term gains into long-term proficiency.

Educational climate and learner psychology

Psychological safety was deliberately cultivated through pre-briefing, supportive debriefing and the egalitarian near-peer relationship. Managing acutely ill patients is intrinsically high-stress, and traditional clinical settings where errors can harm patients often magnify learner anxiety. By contrast, the simulation environment offered a controlled habitat where students could practise and reflect without fear of adverse consequences. Pre-briefings clarified learning objectives, emphasising low-stakes exploration rather than summative judgement, while near-peer facilitation further lowered hierarchical barriers and encouraged open questioning [41]. As tutors were only slightly senior, they understood common anxieties and delivered unbiased, constructive feedback; this social congruence reinforced learner confidence and prompted active participation. Collegial debriefs normalised difficulty, framed mistakes as learning opportunities, and thereby reduced psychological obstacles to taking decisive action during emergencies.

Transformative learning, as articulated by Mezirow and later expanded with Cranton, is “the process of using prior interpretation to construe a new or revised interpretation of the meaning of one’s experience as a guide to future action” [42,43]. Central to this process are the elements of critical reflection, open dialogue and lived experience, which help learners reinterpret existing assumptions [44]. Psychological safety dovetails with this model: when clinical training environments foster a sense of safety, learners feel free to acknowledge uncertainty, voice gaps in their knowledge and practice new skills. Designing simulation with explicit attention to psychological safety is therefore critical for maximising educational impact [22].

Economic viability and institutional impact

Near-peer teaching uses trained senior students or junior clinicians to teach juniors under faculty oversight. The model lifts the knowledge gained per student for each faculty member. One faculty hour supervises several near-peer tutors running parallel small-group sessions. Learners gain more contact time per faculty hour, and the cost per learner falls. Substituting one faculty instructor with one trained near-peer cuts hourly delivery costs by around 60% for the same session length and objectives [45]. At the programme scale, the approach lowers the marginal cost of expanding cohorts and makes simulation and skills teaching financially sustainable.

The model also improves institutional productivity. Senior academics gain protected time for high-impact tasks: grant application, research, curriculum design, accreditation, assessment blueprinting, and staff mentoring. This redeployment lifts quality and reputation. A key risk of this teaching method is quality drift. To mitigate this, faculty must ensure that near-peer tutors are well prepared and maintain teaching standards. Preparation may include brief workshops and meetings prior to each session. Standardised slide shows, teaching manuals and checklists should be provided. These measures help maintain teaching quality and consistency across the sessions.

Strengths and limitations

This study has several strengths. It addresses a critical gap in undergraduate medical education by implementing an acute-care training intervention early in the curriculum, and it evaluates the impact on both knowledge and confidence using a pre-/post-design. The use of a near-peer, simulation-based format is innovative in the pre-clinical context and has proved to be a practical way to teach skills. Such skills are otherwise difficult to gain before rotations. The program achieved a 100% response rate among participants, suggesting strong engagement and the feasibility of our approach. Additionally, our assessment tools (knowledge test and confidence questionnaire) were content-validated and demonstrated good reliability (Cronbach’s alpha 0.85-0.89). Furthermore, the alignment of our course with identified needs (e.g., improving acute-care preparedness as highlighted by prior studies) suggests the intervention was well‑aligned to a recognised deficiency, thereby potentially producing meaningful educational value.

This study is constrained by several methodological limitations typical of SBME research: absence of a control group, reuse of identical pre- and post-tests, single-site design, and lack of long-term follow-up. Given the small student cohort, this study cannot offer definitive conclusions but does highlight consistent trends. In order to confirm and generalise these findings, a multi-centre, controlled longitudinal study that ideally employs alternate assessment tests would be required.

Future research

A prospective, longitudinal evaluation of SAAP graduates as they progress through clinical clerkships and into postgraduate training would allow objective assessment of whether the early gains in knowledge, confidence, and crisis-management behaviour translate into sustained performance advantages and reduced anxiety during real-world emergencies. Concurrently, randomised or quasi-experimental studies that directly compare near-peer-led simulation with faculty-facilitated SBME, while controlling for scenario fidelity and contact time, are essential to delineate relative educational efficacy, tutor development benefits, and cost effectiveness. Such evidence would inform optimal allocation of institutional resources and guide curricular policy on scalable acute-care training models.

## Conclusions

A near-peer facilitated, simulation-based acute-care course can be integrated early in the medical curriculum with demonstrable benefits in knowledge and confidence. These findings support broader adoption of vertically aligned SBME to meet competency frameworks set out by the GMC. Investing in simulation and near-peer teaching can produce graduates ready to manage acute illness from day 1. Each additional graduate who can recognise and act decisively in the first critical minutes of deterioration represents an immediate gain in patient survival and system resilience. Early near-peer simulation converts novice observers into confident first responders, capable of decisive action during the golden minutes of clinical decline.
